# Person-centred integrated primary care targeting patients with chronic conditions and low socioeconomic status: an interview study exploring experiences from healthcare providers and patients

**DOI:** 10.1080/02813432.2026.2734060

**Published:** 2026-10-29

**Authors:** Hester E. van Bommel, Lianne C. G. Roelofs, Tina C. Mantel, Maria E. T. C. van den Muijsenbergh, Jako S. Burgers, Erik W. M. A. Bischoff, Tessa van Loenen

**Affiliations:** ^a^ Department of Primary and Community Care, Radboud University Medical Center, Research Institute for Medical Innovation, Nijmegen, The Netherlands; ^b^ Dutch Centre of Expertise on Health Disparities, Pharos, Utrecht, The Netherlands; ^c^ European Forum for Primary Care, Utrecht, The Netherlands; ^d^ Dutch College of General Practitioners, Utrecht, The Netherlands; ^e^ Department of Family Medicine, Care and Public Health Research Institute, Maastricht University, Maastricht, The Netherlands; ^f^ Department of General Practice, Erasmus MC University Medical Center, Rotterdam, The Netherlands

**Keywords:** Person-centred care, chronic care, low socioeconomic status, general practice, Netherlands, health disparities

## Abstract

**Aim:**

To explore how patients with chronic conditions and low socioeconomic status (LSES) perceived a person-centred integrated-care (PC-IC) approach, developed in co-creation with these patients, general practitioners (GPs) and general practice nurses (PNs).

****Methods**:**

We conducted semi-structured interviews with 18 patients, 2 GPs and 12 PNs who had worked with the PC-IC approach. The approach was developed to tailor primary care for patients with chronic conditions and LSES. It consisted of multiple consultations for a stepwise approach and visual tools. GPs and PNs received training focused on relevant skills before applying the PC-IC approach in practice. The aim of the approach was to adapt care to the context, needs and abilities of these patients.

**Results:**

GPs and PNs reported positive experiences and greater job satisfaction with the PC-IC approach, perceiving that patients felt more heard and engaged. The visual tool supported dialogue, helped to build trust and improved understanding of the complex needs of LSES patients beyond their chronic conditions. They recognised a need for closer collaboration with social care. Patients appreciated being seen more as individuals rather than merely as chronic patients, but noticed limited changes in their care.

**Conclusion:**

The new PC-IC approach with the visual tool for patients with chronic conditions and LSES is positively assessed by healthcare providers (HCPs) and patients. It increases awareness of relevant issues related to patients’ social context among both HCPs and patients. Improving interdisciplinary collaboration with social domain will even further strengthen PC-IC for patients with chronic conditions.

## 1. Introduction

In many countries primary care for patients with chronic conditions is organised through single-disease chronic management programmes, which provide high quality care through highly structured and uniform protocols. However, this may result in overtreatment of patients whose conditions are stable, while patients with more complex or multidimensional care needs may be undertreated [1]. Person-centred and integrated care (PC-IC) for patients with chronic conditions is a promising alternative to single-disease management in primary care. It offers a potential solution to the rising number of patients with multimorbidity (i.e. two or more chronic conditions), the growing demand for chronic care and the increasing workload in healthcare [2,3]. The PC-IC approach may be especially important for patients with a low socioeconomic status (LSES), defined as having a basic or lower vocational education [4]. They often develop chronic conditions at a younger age and have higher rates of multimorbidity than those with higher socioeconomic status [5]. They may also face social problems (e.g. financial insecurity, unemployment and loneliness) that negatively impact their health and quality of life [6], and difficulties accessing healthcare, especially due to limited health literacy and complex psychosocial circumstances [7,8].

To reduce the burden of chronic diseases, a PC-IC approach may improve patient outcomes by shifting the focus from single-disease management to person-centred, tailored care of individuals and their context. A pilot study revealed that the initial PC-IC approach was not positively assessed by LSES patients and their healthcare providers (HCPs), i.e. general practitioners (GPs) and practice nurses (PNs). The approach did not adequately match the communication needs and consultation practices of the participating patients and HCPs [9]. This prompted a co-design process with patients with LSES and HCPs in which the seven-step structure of the initial PC-IC intervention developed by Raaijmakers et al. [10], was kept, but the content, materials and preconditions were redesigned for better alignment with LSES patients [11]. As part of this process, the visual tool was previously developed to support the communication between patients and HCPs, particularly for patients with difficulties in reading, writing or verbally expressing concerns and preferences, and for patients experiencing problems across multiple life domains.

This qualitative study, embedded within a mixed-methods cluster randomised controlled trial (cRCT), aimed to explore how patients with LSES and HCPs involved, perceived the use of this new PC-IC approach and to identify their needs for optimising the approach for broader implementation in primary healthcare.

## 2. Method

### 2.1. Design

From January 2023 till May 2024, a cluster randomized controlled trial (cRCT) took place to assess the effectiveness of the PC-IC approach in general practice for patients with a chronic condition and LSES (ClinicalTrials.gov ID NCT05972031).

As part of the effectiveness evaluation, we conducted a qualitative study using semi-structured interviews with GPs, PNs and patients with LSES to explore their experiences with the PC-IC approach.

### 2.2. Setting and study population

In the Dutch primary care setting, so called ‘chronic care consultations’ are structured consultations for patients with chronic conditions, such as diabetes, cardiovascular disease or chronic obstructive pulmonary disease (COPD). These consultations are often conducted by PNs (under GP supervision), within primary care management programmes, which contain strict protocols for disease management. A consultation lasts about 30 min on average. In the Netherlands, chronic care in primary care is structurally supported through contracts and funding arrangements with healthcare insurers, which may differ from healthcare systems in other countries.

Eleven general practices in rural and semi-urban areas of the Netherlands, supported by six regional primary care cooperatives, replaced their standard disease management programmes for type 2 diabetes, cardiovascular disease, COPD and asthma with the PC-IC approach for LSES patients during their ‘chronic primary care consultations’. These eleven practices were allocated to the intervention arm of the cRCT through randomisation from a total of 22 participating general practices. The experiences reported here refer only to the practices that implemented the intervention, not to the practices that continued care as usual, i.e. the standard disease management programmes. LSES was defined as having completed no more than post-secondary vocational education level 1 (or equivalent), and/or indicating a need for help with reading in the self-reported baseline measurement at the beginning of the cRCT study. Eligible patients with one or more of the chronic conditions, low educational level and informed consent were recruited and followed for 12 months, starting between January and May 2023. Participants received information about the study before participation and gave informed consent. We adapted the information to make it understandable for patients with limited health literacy. Prior to the intervention participating GPs and PNs received extensive training and supporting materials to implement the approach. The training lasted 6 h in total, divided over two days with one month in-between. During this interval, participants could practice the acquired skills and discuss difficulties at the last training session. The training was facilitated by an experienced General Practice trainer together with a simulated patient. It consisted of lectures as well as exercises for participants to practice communication skills in realistic scenarios.

Participants for the in-depth interviews were recruited at the end of follow-up by the research team using purposive sampling. GPs and PNs were recruited from the participating general practices, aiming to include at least one GP and one PN per practice, with attention to variation across urban and rural practices. Patients were recruited with similar sampling strategy aiming for diversity in age, gender, migration background, geographic location and general practice.

The Research Ethics Committee of the Radboud University Medical Center granted ethical approval exemption in accordance with Dutch national legislation (registration number 2022–15786).

### 2.3. The new PC-IC approach

The HCPs in this study applied a new person-centred integrated care approach that built further upon a recently developed PC-IC approach for patients with chronic conditions in primary care (see Figure 1) [10].

**Figure 1.**
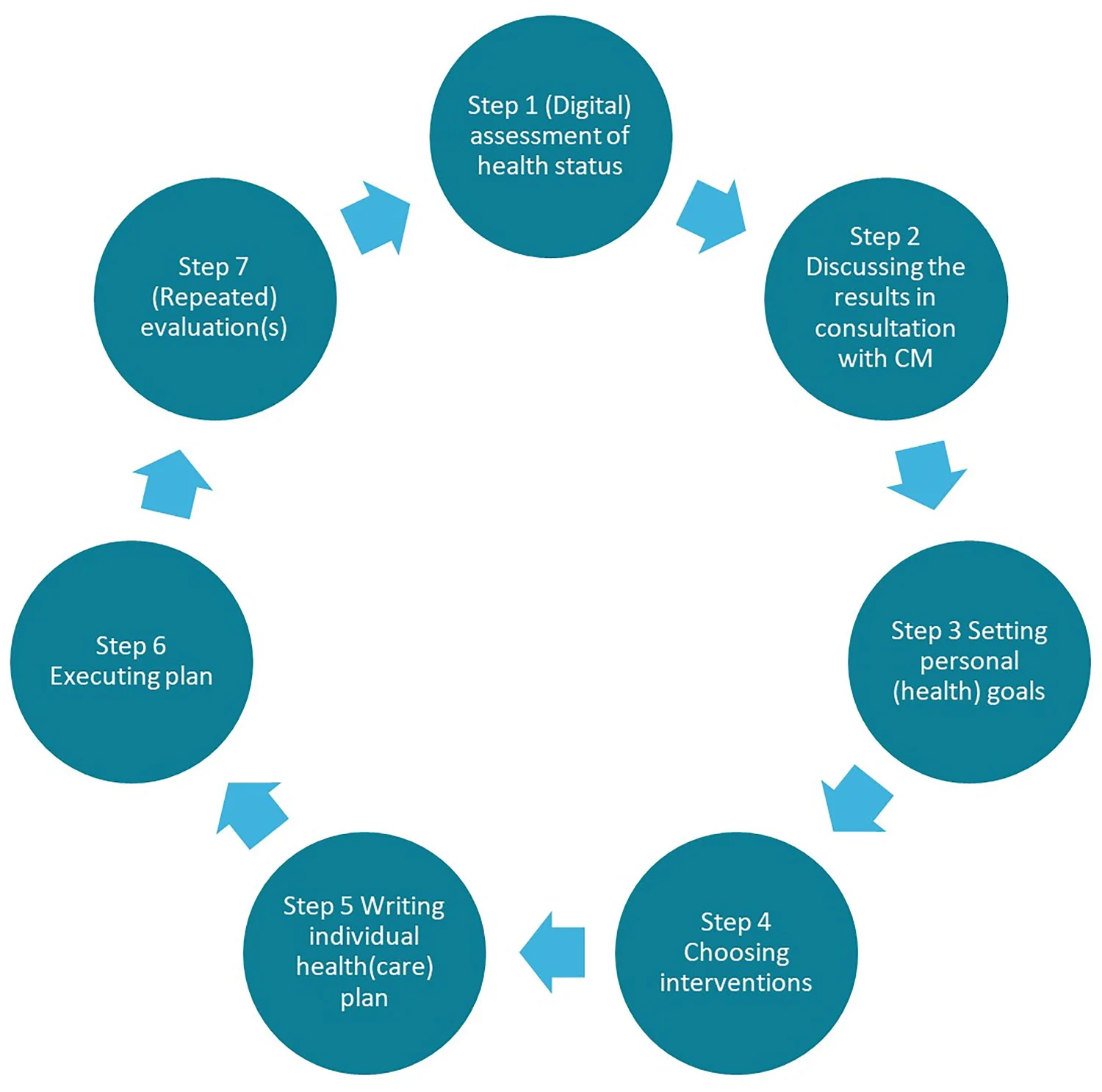
Schematic representation of the PC-IC approach for the management of patients with chronic diseases and multimorbidity in general practice [10].

This approach was redesigned to meet the needs of LSES patients, through co-creation sessions with stakeholders (see Table 1) [11]. While the original model’s seven cyclical steps were retained, its content, materials, key points of attention and organisational prerequisites were revised and expanded (see Figure 2 and Appendix 1). To support the patients in discussing with their HCP the items relevant for them, a visual tool was previously developed in a co-creation process with patients and HCPs (see Figure 3). This tool was designed to facilitate accessible and understandable communication, and aimed to help patients articulate concerns, priorities and goals within the consultation and thus enhance shared understanding. Our research on the development of the tool has been published elsewhere [11]. This new approach was subsequently introduced during training sessions for GPs and PNs [11]. These sessions focused on person-centred care and on how to initiate a person-centred conversation. Special attention was given to accessible and understandable communication with patients who are low literate or have complex social problems, as well as to interprofessional collaboration. HCPs also practiced how to use the visual tool in a flexible manner during consultations, tailored to patients’ individual needs and contexts.

**Figure 2.**
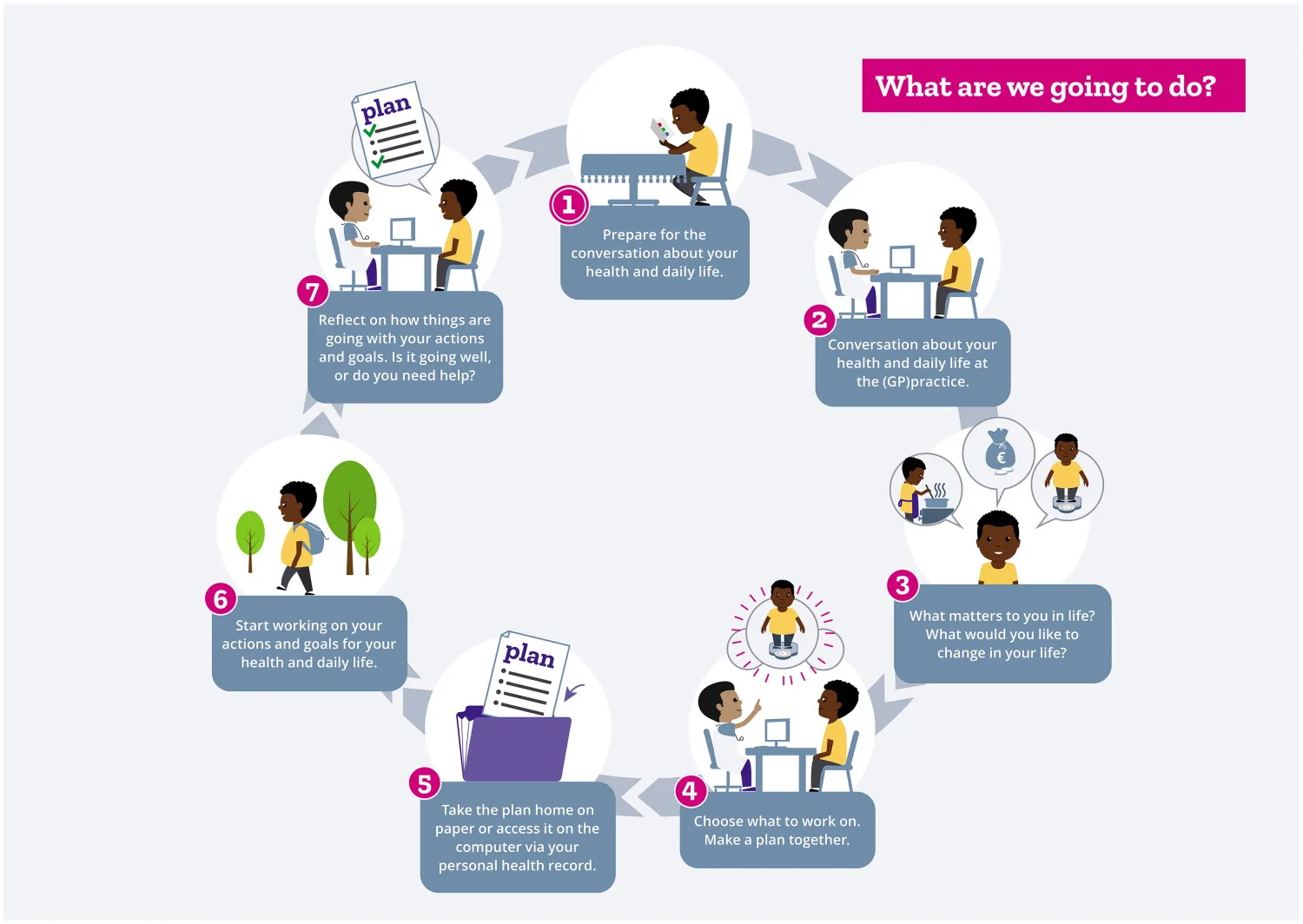
Schematic representation of the PC-IC approach with seven steps for the management of patients with chronic diseases and multimorbidity in general practice. This representation clearly illustrates what the patient may expect (see also Appendix 1).

**Figure 3.**
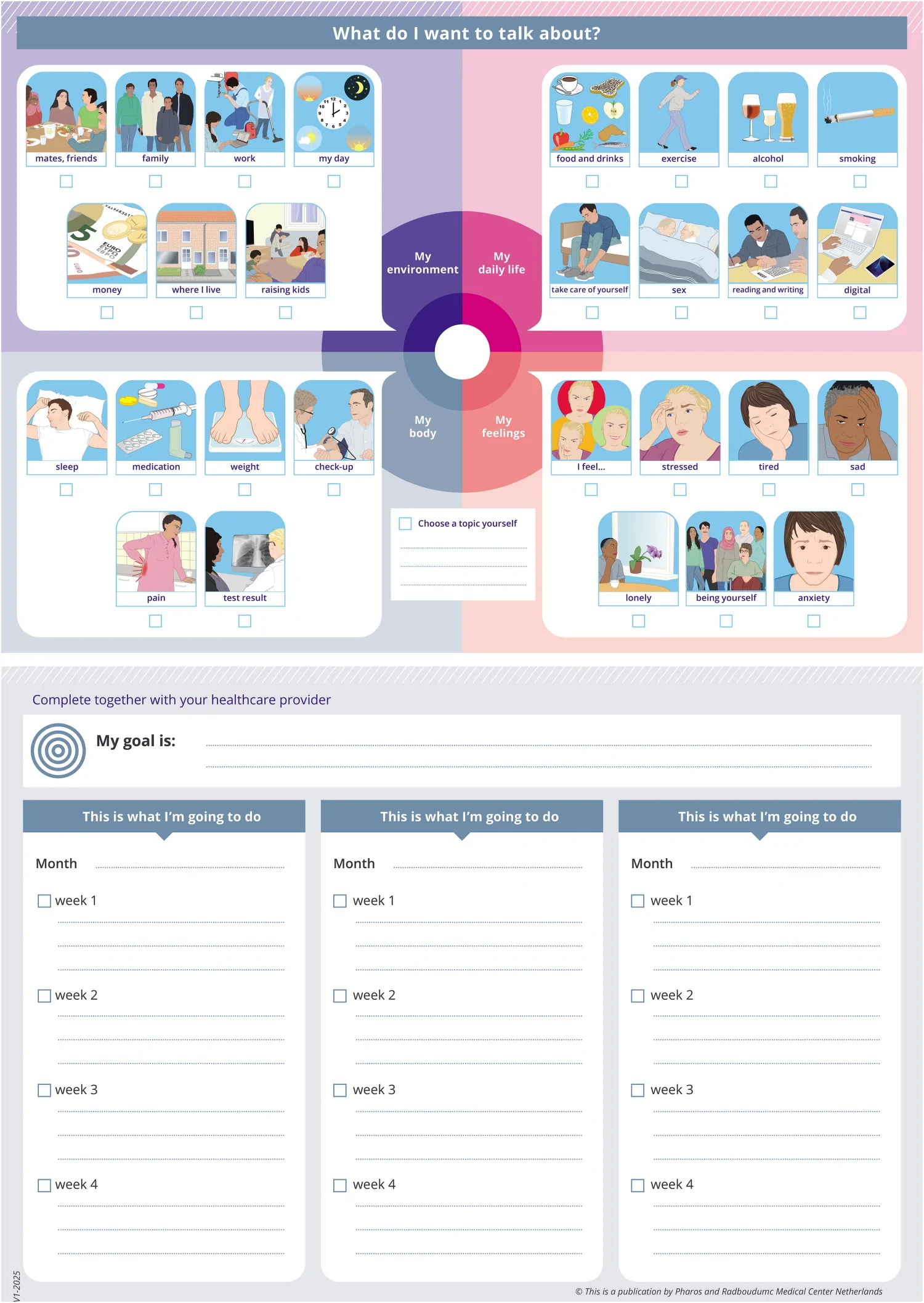
Visual person-centred conversation map covering multiple domains to prepare and support in the PC-IC conversation.

**Table 1.** Adaptations in the seven steps of the PC-IC approach for LSES patients with chronic conditions.

Step	‘Generic’ PC-IC approach	Adjusted PC-IC approach LSES
1	Digital assessment of health status at home.	Patients prepared at home using the visual person-centred conversation tool (covering ‘my environment’, ‘my daily life’, ‘my body’ and ‘my feelings’). GPs and PNs used the tool during the consultation to guide the conversation and set expectations.
2	Discussing the results (from the assessment) in consultation with case manager.	During the consultation, the patient and GP or PN used the visual PC-conversation tool with its four domains, while the GP or PN communicated clearly and empathetically, addressed low health literacy, used visual materials to support comprehension, considered the patient’s social context (e.g. stress, migration, poverty) and applied the teach-back method.
3	Setting personal (health) goals	Together with the patient, the GP or PN identified what matters most in their life and what changes they wish to make, taking into account their social context, while reviewing the different domains to explore possible entry points and jointly establish priorities.
4	Choosing interventions	The GP or PN and patient collaboratively set priorities and developed a realistic action plan that considered the patient’s skills and abilities, set achievable goals, used accessible support tools and involved other professionals when needed.
5	Writing individual health (care) plan	The plan is shared with the patient – either in printed or digital form – using clear, plain language.
6	Executing plan	The GP or PN supports the patient in implementing the agreed steps for health improvement, maintains contact to assess what is needed to achieve the goals, and facilitates a warm transfer to a colleague or community intervention when appropriate.
7	(Repeated) evaluations	The GP or PN evaluates progress by discussing successes and challenges, regularly asking what support the patient needs. The organisation provides case-based discussions to GP and PN to structurally embed this approach in practice.

The GP and PN are both responsible for coordinating care, and managing the patient’s social support. In this study, PNs were predominantly responsible for implementing the intervention, as in the Dutch primary care system PNs primarily manage patients with chronic conditions under supervision of a GP. The GP sees the patient with a chronic condition annually to evaluate the care. Central to the PC-IC approach is the PC-conversation (Steps 1 and 2) that addresses the patient’s chronic conditions as well as broader psychosocial needs and concerns, supported by a visual conversation tool (see Figure 3). The visual conversation tool with four domains – ‘my environment’, ‘my daily life’, ‘my body’ and ‘my feelings’ – enables open discussion of all relevant life areas, including sensitive issues, such as financial strain, stress and literacy challenges. It helps shift the consultation focus from disease management to the patient’s overall well-being.

Steps 3–7 concern setting patient-centred goals, creating and documenting a tailored plan, taking action and evaluating progress with adjustments as needed. To prepare for this way of working, GPs and PNs received training to develop the necessary skills for providing PC-IC to LSES patients. This included practising communication techniques, applying the teach-back method [12] and learning to collaborate across care domains. After four months, case-based discussion meetings were held with participating GPs and PNs to exchange experiences, explore challenges in applying the adapted PC-IC approach and jointly develop strategies for person-centred care in complex situations.

### 2.4. Data collection

Individual in-depth interviews were conducted using a semi-structured topic list – one for GPs and PNs and one for patients (Appendices 2 and 3). The topic lists were developed by two researchers (TL and HB). The topic lists were based on literature on person-centred care [6,13], the house of care model [14] and the access-to-care framework [15], which emphasises that access includes both availability and acceptability of care.

Key elements and themes were extracted from this literature and further informed by expertise (HB) from Pharos, the Dutch Centre of Expertise on Health Disparities. For patients, interview topics covered experiences and communication with their GP or PN (e.g. clarity, trust, the PC-IC approach, its barriers and facilitators and use of the developed tools). For GPs and PNs, topics included these aspects of the PC-IC approach as well as collaboration with other providers and domains, shared decision-making, provision of easy to understand and accessible information, experiences with PC-training and use of the developed tools. The topic guide was refined based on the insights gained after the first interviews [16,17].

The interviews were conducted by several researchers (HB, LR and DD). Data collection continued until data saturation was reached, defined as the point at which no new information emerged within each category of the framework analysis [18].

### 2.5. Data analysis

The interviews were audiotaped, transcribed ad verbatim by a professional human transcription service contracted by Radboud University Medical Center, and coded by researchers from the research team (HB, LR and DD) independently to ensure coding validity. ATLAS.ti (ATLAS.ti Scientific Software Development GmbH, version 24) was utilised for this coding process. Framework analysis was used as the primary analytic strategy to systematically analyse participants’ experiences in relation to the PC-IC approach [6,13–15]. The initial code tree was deductively informed by the steps of the cyclical PC-IC approach (Figure 1). Subsequently, inductive open coding was used to identify additional themes and insights emerging from the data. The first four interviews were independently coded by three researchers (HB, LR and DD). Subsequently, consensus meetings were held to discuss differences in coding and refine the code tree. The remaining interviews were then coded using the revised coding framework. Any coding uncertainties arising during the analysis were subsequently discussed within the research team and resolved through consensus. After coding, the data were thematically organised and conceptual themes were identified to structure and connect the codes (HB and TM).

The study was reported according to the standards for reporting qualitative research [19].

### 2.6. Ethics

Written informed consent was obtained from all participants prior to data collection. The medical ethics review board of the Radboud University Medical Center judged that ethics approval for the study was not required under Dutch National Law (registration number: 2022–15786).

All signed informed consent forms and audio recordings are securely stored on a protected server at Radboud University Medical Center. All study data are pseudonymized to ensure participant confidentiality and privacy.

The audio recordings were transcribed verbatim by a professional human transcription service contracted by Radboud University Medical Center. The transcription process was conducted in accordance with the institution’s applicable data protection and confidentiality requirements.

## 3. Results

### 3.1. Characteristics of the participants

In total, 18 patients with a chronic condition and 14 HCPs were interviewed (Tables 2 and 3). Half of the patient participants were aged 65 years or younger, and nine had multimorbidity. Fourteen reported needing occasional or frequent assistance in understanding medical brochures. Among the patient participants, nine did not have paid employment. Of these, five had been declared unfit for work, one was unemployed, one had taken early retirement and two were housewives. The remaining participants were retired, with one engaged in volunteer work. One patient had a migration background (Table 2). Regarding the HCPs, 12 were employed as PNs and two as GPs (Table 3).

**Table 2.** Characteristics of patients.

Characteristics patients (*N* = 18)		Number
Age	50–55 years	3
	56–65 years	6
	66–75	6
	>75	3
Sex	Male	6
	Female	12
Migration background	Yes	1
Educational Level*	*Primary education*	*2*
	*Primary/preparatory vocational education*	*16*
Employment	Paid work	–
	Housewife/man	2
	Unemployed	1
	Retirement	9
	Retire and volunteer work	1
	Declared unfit for paid employment (80–100%)	5
Reading ability	Requires no assistance in understanding medical brochures	4
	Requires occasional assistance with comprehending medical brochures	11
	Requires frequently assistance with comprehending medical brochures	3
Chronic conditions	Only diabetes mellitus	3
	Only COPD/asthma	3
	Only cardio vascular disease	5
	Multimorbidity (two or more chronic diseases)	7

COPD: chronic obstructive pulmonary disease.

* In italic the number of patients with lower socioeconomic status.

**Table 3.** Characteristics healthcare providers.

Characteristics healthcare providers (*N* = 14)		Number
Age	30–40 years	1
	40–50 years	4
	50–60	7
	>60	2
Sex	Male	2
	Female	12
Function	Practice nurse	12
	General practitioner	2
Practice	Solo	3
	Duo	2
	Group	9
Work Experience	0–5 years	5
	5–10 years	2
	>10 years	7

### 3.2. Results in-depth interviews

The interviews with the GPs and PNs took 45–60 min each, and were held online *via* Microsoft Teams or face-to-face at the general practice. The interviews with patients took 21–55 min each, and were held face-to-face at the patients home.

We identified five themes reflecting the experiences of patients, GPs and PNs with the newly adapted PC-IC approach. The first theme, describes experiences during the study with the approach including the visual PC-conversation tool, shared goal setting and engagement. The second theme highlights the importance of accessible and understandable communication and trust. The third addresses interdisciplinary collaboration, particularly when patients present with complex conditions involving both medical and social problems. The fourth and fifth themes concern preconditions and job satisfaction obtained.

#### 3.2.1. HCPs’ and patients’ experiences with the PC-IC approach including the visual PC-conversation tool

##### GPs’ and PNs’ experiences

Most HCPs viewed the PC-IC approach positively as they gained deeper and new insights into patients’ living conditions, even after years of treatment. Although HCPs are generally trained to take a holistic view of their patients’ symptoms, in the context of chronic care, the focus used to be often narrowed specifically to the chronic condition itself.

The use of the PC-conversation tool stimulated broader discussions and helped to clarify patients’ actual needs, and allowed for better differentiation in care. The PC-conversation tool led to a different approach. Non-medical topics, such as financial difficulties, family or relationship issues and stress, were frequently addressed. They observed that it broadened the scope of discussions and contributed meaningful value to the consultation process.

What we really want—and what I aim for in consultations—is to assess what this specific patient truly needs fromme. This is not always another pill, a lecture, or another blood test showing poor results. It requires a broaderperspective, considering the patient’s social context and how primary care can influence it to ultimately improvechronic care. (HCP11-GP)

Among the majority of professionals, the visual tool was placed at the desk as a standard setup, which facilitates patient engagement with a wider range of topics. HCPs reported that the tool encouraged patients to reflect on issues they might not otherwise have raised independently and helped uncover longstanding concerns that had not previously surfaced during consultations. It also encouraged patients to reflect more consciously on their health and life circumstances.

I see many patients who struggle to make progress, and through different conversations, it becomes clear that theyface numerous underlying problems. Then it makes sense why they cannot improve, why we keep hitting the samebarriers, and why chronic care remains ineffective, leading to complications. We need to address this differently.This approach allows for a more precise assessment of what each person specifically needs. For most chronicpatients, these needs differ. Some do not require anything from me at all. Others need something I cannot provide,such as support from a social worker. Some do need my help. This differentiation is valuable, and we can now see itmuch more clearly. (HCP11- GP)

The PC-conversation tool is always present—it is standardly on my desk. Approximately once every three months,I see the patient again. I notice in the journal that I have ended up with that, and then I check back on it. I ask, “Isthere anything else you would like to discuss?” (HCP1-PN)

A few HCPs did not use the tool and indicated that they already knew the patients and their families well or found it difficult to use a tool with pictures in consultation. They had the experience that patients started pointing out all the pictures, which made the situation somewhat chaotic and made it challenging to address all the intended topics. For them, integrating these broader discussions also required more time.

The HCPs mention various strategies for offering the PC-conversation tool to patients. In some cases, it was sent or handed out prior to the consultation, enabling patients to reflect on their concerns and priorities in advance. In other cases, the tool was first discussed during the consultation and then given to patients to further reflect on at home before the next appointment.

So people don’t always immediately know what to bring up, but I always gave them the conversation tool to take home. […]. I gave it as a sort of homework, encouraging them to think about it or note down topics they wanted to discuss at their next appointment. Sometimes, patients feel put on the spot when they see the conversation tool, thinking they have to come up with everything all at once. Taking it home gives them more time to reflect. (HCP3-PN)

##### Patient experiences

Many patients reported that consultations were enriched through the PC-conversation tool, compared with prior practice. They felt encouraged to discuss not only their illness, but also broader aspects, such as work, family, sleep, nutrition, weight, smoking, medication and exercise. Some patients appreciated that their HCP consistently asked about their overall well-being, and some also noticed a positive change in the consultations, observing that their HCP took more time for them.

Because with your doctor you always think, no time or I only come for that. I can’t address another issue there. For many people it would be easier, I think. […] Well, to start the conversation. (P7-female 77 y)

Patients reported that the visual PC-conversation tool is a good method to start or prepare for a consultation. It covered new topics that had not been discussed in previous consultations. It helped to bring up different topics other than their disease. One patient also mentioned that it felt like a relief to share concerns, such as family problems or past events. Most patients indicated that the topics included in the conversation tool were relevant and that they felt comfortable discussing them. One patient emphasised that it is not just about the disease you have, but also about how you feel.

There were a few more conversations brought to the table this time, because they were indicated in the images. So things naturally became a bit broader. I am someone who is quite good at talking anyway, but now we also spoke about family life, about this [points to image], about smoking. (P15- female 63 y)

Some patients received the PC-conversation tool during consultations, either to prepare for their next appointment or to prompt additional topics during the consultation. Experiences varied: while some found it useful – particularly when their HCP referred back to it – others noted it was not addressed in follow-up consultations. A few initially felt uncomfortable using the tool, but later found it helpful for discussing concerns and thoughts.

Yeah, if you want to bring something up yourself, it doesn’t always occur to you when you’re sitting there. But then, if you’ve check-marked it and she asks, like, ‘What about family?’ or ‘What would you like to share about family?’, I like that. Because sometimes you forget things as you get older. For example, if you’ve check-marked four or five things, I forget them once I’m there, you know? (P7-female 77 y)

While some patient reported these positive experiences, others felt that consultations still predominantly centred mainly on illness and treatment, often attributing this to lack of time. Some indicated that they did not require a tool to raise relevant topics, having found solutions independently or not wishing to change their behaviour. In certain cases, this led to frustration when recurring themes (e.g. smoking) were repeatedly addressed. Other topics, such as sexuality, were considered too private to discuss with an HCP.

A few patients initially did not recall the tool but responded positively when shown it during the interview, highlighting its clarity and relevance. Some even expressed interest in bringing the tool to future consultations with their PN.

Both HCPs and patients emphasised that the visual tool helped to broaden the scope of consultations and added meaningful value to the care process. By systematically including a wide range of topics, the tool encouraged discussion of topics and concerns that might otherwise have remained unspoken. This not only increased patients’ awareness of their own health trajectory but also facilitated more comprehensive discussions about the interaction between medical and non-medical issues in chronic care. Some HCPs mentioned patients usually do share the physical symptoms, but it is precisely the non-medical topics that are now being discussed and had an impact on the chronic condition.

Overall, patients tend to respond well to the use of the conversation card. They appreciate the opportunity to explore certain issues in greater depth. As previously mentioned, the card helps to surface topics that are either difficult to discuss or have been a source of concern for some time.

One example involved a patient who was living with his daughter-in-law. Over time, their relationship had becomeincreasingly strained. Such matters are not typically shared unprompted during consultations. However, throughthe use of the conversation card, this underlying source of stress was revealed—a stressor that could beaddressed in a meaningful way. This prompted a detailed discussion, and the patient expressed a desire to activelyengage with the issue. He subsequently sought support to help him manage the situation, particularly given thatthe living arrangement was expected to continue for some time. While such situations are rarely resolved quickly,the patient’s well-being improved following this intervention. It is likely that, without the conversation card, thisissue would not have been brought to light. (HCP4-PN)

##### Different experiences of setting treatment goals by HCP and patient

The HCPs experienced that the PC-conversation sometimes was helpful to explain that the treatment a patient wanted was not the most appropriate one at that moment. Some HCPs also noted that goal setting seemed more meaningful because of the person-centred conversation, as already some topics were discussed together. Having a PC-conversation also allowed other concerns or issues to surface, upon which the HCP could set appropriate goals or advice with the patient. Examples included referring patients to ‘coping with stress training’, engaging social work to address social problems or referring them to walking groups or participation groups in the context of loneliness or day care.

You really create much more together that way. Usually, you just say, ‘Go cycling or walking for 30 minutes a day, start eating healthy, and I’ll see you again in three months’. As a healthcare professional, you’re already thinking ahead in that plan. But when you start setting those goals together, it changes things—we often tend to fill in a lot ourselves. By setting goals together with the patient, you give some of that control back to the patient. (HCP12-GP)

Patients, on the other hand, had a different experience; they generally did not recall setting or agreeing on goals during the consultations describing the interactions as largely one-way. HCPs mainly advised or instructed them according to the patient, such as eating more vegetables or increasing social contact, without tailoring these goals to individual needs or abilities. While a few patients reported setting shared treatment goals with their HCP, others noted that their HCP did not talk about goals or plans at all. Some patients expressed reluctance to set goals, either to avoid disappointing their HCP or because they knew that they would not follow through.

I think we actually do it together. Sometimes she says something and sometimes I ask myself: can I do this or can I do that? (P15- female 63 y)

A few HCP documented the goals directly on the visual tool together with the patient. However, most did not use the visual tool for this purpose without providing a reason for not doing so. The patients reported various experiences: some had not seen or used the tool for goal setting, others felt it was unnecessary as they believed that they could remember the goals without assistance of the tool. The HCP recorded the goals in the digital patient medical record rather than on the tool itself.

##### More self-management and involvement by patients own treatment

The HCPs experienced more self-management among their patients through this PC-approach. By allowing patients to tell their own story using the conversation tool or by asking questions, patients also indicated that, for example, their complaints or pain are part of it, but are not actually the biggest problem they suffer from. Previously, this would not have come to the surface. By using the PC-conversation tool, patients were encouraged to take more control or were actively involved in their treatment. The HCPs also reported that they had to let go more often, refraining from immediately solving the problems., This approach represented a new way of working for them. Some HCPs indicated that taking more time during the consultation for the PC-conversation made them feel less rushed and allowed them to lean back more. This created space for more in-depth conversations, enabling patient to speak more freely. Patients even opened up about topics they had never discussed before, often due to feelings of shame. For example, one of the PN said she had been treating a patient for years, but had never known she was forced to sleep on the sofa in the living room because her bedroom and entire house were full of stuff and she had nowhere to go. Once this problem was addressed and the patient received appropriate support, her diabetes also became better controlled.

That you really make people more aware of certain issues that they wouldn’t necessarily think of themselves. And by being able to use these conversation cards, you notice that people come up with other things that you don’t normally just think about and that you find out that there is more going on than you actually know, that they want to tell you. That card triggers people to be more open and because they are more open and can work on things, they are more aware of their own process. So I just notice that shifts are taking place. And so people actually want to do their best even more because of that. […] You get much more out of it than what’s in it actually, very briefly. Both for ourselves and for the patients. (HCP4-PN)

#### 3.2.2. The importance of accessible and understandable communication and trust

##### Providing understandable and accessible information

Most patients reported positive experiences with understandable information from their HCP. However, some described difficulties in understanding diagnoses or treatment instructions, which occasionally led to incorrect medication use or feelings of not being heard.

I told her [the HCP] about my legs, because you can see it—they’re all purple. She told me to use the treadmill, but I thought, that’s not right, because when I walk a mile, I feel pain in my calves. There’s clearly something wrong, and then I have to stop. I wonder, who knows best how it feels? I said I have an injury, but she said, ‘No, you just have to walk; it’s oxygen deficiency in your leg’. And I thought, how can that be? I do go outside sometimes…. (P8-male 65 y)

One patient emphasised the importance of tailored communication, appreciating that the HCP recorded blood pressure and weight because the patient often forgot. At times, patients withheld information, citing reasons, such as privacy, doubts about the HCP’s ability to help, self-reliance or feeling patronised.

Yes, she [HCP] always makes me feel good. She listens to me and she knows how to answer and what I should do and how. And she also says, you know, if it doesn’t work out, call me. (P10-feamle 65 y)

Some HCPs reported that this approach and the use of visual aids made them aware that their own explanations were sometimes too difficult. Another HCP said that it helped her to check whether patients understood her, which led her to give clearer explanations, write down advice and use the teach-back method more often.

Accessible and understandable communication is not ensured for all patients *via* the digital medical portal, as some experienced access barriers and prefer direct contact by email or phone. As a result, important information about medical results and follow-up steps was often not clearly conveyed or understood. Furthermore, many patients noted that when healthcare professionals fail to return calls as agreed, they do not initiate follow-up themselves, assuming the provider is too busy. Some patients reported still awaiting a call to schedule a new appointment.

I can’t get the digital medical record to open. I know I should ask my PN for help with that. Logging into the healthcare domain is fine, that part works, but then you have to send an email, log in again, request a code—and by the time you use it, the code has already expired. And I respond to that. Because every time I got a reminder of you haven’t read your mail [in the digital medical record] yet. And then the third time. I said, ‘I can’t read that’. It used to be much simpler, if you had to have blood tested and the doctor got the result, you got a phone call. (P11-male 70 y)

##### Mutual listening and building trust

Both HCPs and patients highlighted the importance of mutual listening for accessible and understandable communication. Patients valued when their HCPs took time, showed genuine interest in their well-being and created a safe environment to discuss a wide range of issues. HCPs reported similar experiences. Several noted that they had become more aware of the importance of truly listening rather than prematurely drawing conclusions. As a result, some began to take more notes, for example, regarding medication, and made a greater effort to summarise information for the patient or to check whether the patient had fully understood what had been discussed.

We are often quick to fill in, of you have to do this and you have to do that. But sometimes we don’t listen well enough to people, of what indeed is going on. Especially so indeed really listening and really asking what is there at this moment that really concerns you. Why is it going the way it’s going? What is holding you back? There is something. And then sometimes you need a few consultations to find out. And that’s okay, because then you just invite them more often instead of every three months you do it every two weeks. (HCP12-GP)

HCPs emphasised that trust is essential for effective treatment but must be built over time. They indicated that the PC-approach supports this process by facilitating broader conversations and deeper engagement, thereby strengthening therapeutic relationships. Allowing more time in consultations was considered key to fostering patients’ confidence to express their concerns.

I was actually shocked that people who are refugees […] she told her whole story to me of that flight. And she never did that before. While I was actually quite open to it, but through the pictures, [.] and that you then get a hug at the door, while they have been so sad by telling it again, how important it is. That to me indicated even more how important it is to be open to other things and that for them the picture was suddenly anyway…, because I could communicate reasonably well with that her, she does not actually have a language problem at all, you think. But still through pictures [the conversation] goes much deeper. I was shocked by that. (HCP9-PN)

#### 3.2.3. Interdisciplinary collaboration is needed within person-centred care

Several HCPs reported that the PC-approach required collaboration with social work and other domains, which enhanced interdisciplinary cooperation both within general practice and beyond. At the same time, they found it challenging to address the wide range of topics raised during consultations, noting that a strong professional network was essential. A further concern was the limited or unclear feedback from referral organisations regarding the follow-up support provided to patients.

We also got an overview, one of the GPs made that. A very nice overview. Do you have money problems? Go there. Do your children need a school bag or a bicycle? So do I. That’s also easier to refer.[…] So I refer much earlier, much faster than in the past. Because you also know, for that problem you can go there. I also arrange it. So not leaving an address, because then they do the question do they [patients] actually do it? Do they [patients] understand it? (HCP1-PN)

I sometimes find the feedback from social work a bit lacking. I would like to see a bit more feedback on that. We have someone from social work here at the practice every week, and she also receives patients who have been referred to her by the GP, for instance, or by us. Then she puts out lines to organisations where people can possibly go. But if they are then referred to another organisation, for instance, I don’t get anything [information] in return. (HCP2-PN)

Most patients mentioned that they did not experience any differences in their HCPs’ collaboration with other HCPs since the PC-approach was introduced. According to most patients, their HCP already collaborated with other disciplines, such as mental primary health care. Some patients described that they did not want to consult other professionals besides their own GP or PN, for a variety of reasons. The reasons mentioned were a bad experience earlier in life regarding the discipline, already came to many different HCPs, do not got along with the referred HCP, earlier experiences with expensive costs of care and patients did not feel the urgency of the problem as the HCP did.

#### 3.2.4. Training, less administration and time are the prerequisites for the effective implementation of this PC-IC approach

Before the GP and PN used the PC-approach in practice, they had first undergone training for it. Most of the GPs and PNs found the training of added value and also an eye opener, mainly due to the communication skills that were trained. They recognised that engaging in a broader conversation allows them to better understand the patient’s personal context, needs and motivations, which are essential for truly person-centred care.

For myself, that’s definitely a bit of an eye opener. Again, what I said earlier, that you have to keep that in mind. So all those aspects that are, of course, in addition to the purely medical, which of course we as general practitioners are more concerned with. But that you consider: okay, where does this patient come from? How does he live? Are there other things? What else is going on besides of course purely sec that laryngitis? That is … Why does that patient come with that complaint? It’s no more than getting behind the complaint itself to clarify the request for help. The help question behind the help question. (HCP15-PN)

The GPs and PNs also reported becoming more aware of their own, sometimes medical, language. They indicated that it still requires a different way of working than you are used to. It is additionally challenging to keep working this way. It also has added value to follow this with your whole team.

Some PNs had also learned about person-centred care during their education. For them it took less getting used to this way of working, however, the use of a visual tool to support the communication was new. They indicated that letting go of protocols was more supportive. Many HCPs previously experienced spending a lot of time with processes and protocols. With the PC-approach, the GP and PN experienced that you can achieve more for the patient. They indicated that for this to be implemented properly, the protocols and checklists must be stopped.

Although not all HCPs explicitly mentioned time, it emerged as an important precondition, particularly among GPs. They emphasised the need to allocate sufficient time for person-centred consultations, as these conversations may raise a wide range of topics. In general, PNs tend to have more time available, making this approach easier to implement for them compared to GPs.

But the conversations are truly different. And as practice nurses, we do have the time for them. I know, for example, that my GP colleague also said this approach has given her a very meaningful way of working. But she also said, “I simply don’t have enough time for it—I have to fit everything into ten minutes, while you get half an hour per patient. So I can imagine you’re able to do more with it.” That said, she still applies it a great deal. Just recently, she told me she was wondering why a particular patient was so reserved. She decided to ask a few more questions, and it turned out there was an entire history behind it—poverty, difficult experiences from the past. (HCP12-PN)

#### 3.2.5. Job satisfaction and awareness PC-IC approach

HCPs experienced new insights from working with this PC-approach focusing on LSES patients.

Most HCPs reported increased job satisfaction due to more enjoyable, meaningful and qualitatively different conversations with their patients. These interactions were characterised by greater depth and engagement. Additionally, HCPs experienced this way of working as enriching. They felt able to provide better, and potentially more effective care. One HCP noted that by gaining a deeper understanding of a patient’s social context, she was able to refer the patient more appropriately, contributing to improved health outcomes.

I see patients for whom nothing seems to go right. In those situations, even a small step forward is a major achievement. […] But when a patient says, “This and that is still difficult, but this small part is going better,” it gives me energy too. (HCP11-GP)

Yes, it certainly brings more joy to the work, because this is what you aim to achieve. It aligns with the core intention: to encourage and motivate patients to take care of themselves. Ultimately, that is what it’s all about. (HCP4-PN)

I put in more effort. I invest more energy in the patient. If I notice that they come back to a certain topic, perhaps prompted by a visual aid, and if they had said nothing, the topic would have been closed. But now I notice that I dig a little deeper. How shall I put it? It’s actually enriching that this comes up in the conversation. (HCP1-PN)

HCPs perceived increased awareness of the complex problems many patients face beyond their chronic condition. This shift in attitude encouraged them to explore underlying issues rather than strictly follow checklists, leading to more meaningful conversations, particularly with patients whose care was not progressing well. Such patients often struggle with medical instructions, increasing the risk of errors and poor outcomes. HCPs noted that providing extra attention within a person-centred approach allowed patients to express confusion more openly, fostering mutual understanding and enabling more effective, tailored care.

We are now actually much more aware of where the problem lies. Instead of coming up with some kind of crazy checklist of ‘OK, we must have asked this and we must have probed that and we must have tested that’, this changes the way of thinking and makes it much easier to talk to someone about why it’s not working. And those are of course often the people whose sugar is really sky-high, just not under control. So these are precisely the people who have a lot of complications and require a lot of medical care. (HCP15-PN)

## 4. Discussion

### 4.1. Summary

This study explored GPs’, PNs’ and patients’ experiences with the PC-IC approach developed for patients with LSES. All groups perceived it as valuable for broadening consultations beyond medical symptoms towards a more holistic, non-medical conversation, resulting in more mutual understanding and more tailored care. The findings suggest that the visual tool indeed supported patients in their communication with their primary HCP. It appeared to help patients with chronic conditions and HCPs structure conversations, clarify concerns and needs and in that way it supported mutual understanding. GPs and PNs gained deeper insight into patients’ living conditions – even after years of providing care. The visual tool particularly helped expand the dialogue and prompted patients to reflect on unspoken issues, such as financial stress or social isolation. This supported more meaningful and personalised goal setting. However, some patients felt that goals were still set for them rather than with them, indicating room for improvement in shared decision-making.

GPs and PNs observed an increase in self-management in some of their patients and noted that patients often cited non-medical problems as more burdensome than physical symptoms. Both patients and HCPs felt that the approach facilitated mutual trust, which was seen in both groups as essential for effective care.

A notable finding was the discrepancy between HCPs and patients regarding the content of the conversations. Patients recognised having these conversations but did not experience them as different from usual care. In contrast, HCPs shared cases where the conversation revealed issues they would not have discovered otherwise, leading to a better understanding of the patient and, in some cases, improved management of chronic conditions.

Despite general enthusiasm, some HCPs were concerned about increased workload and patients’ higher expectations for attention. Integrating broader discussions required more time and a shift in professional roles, including resisting the impulse to immediately resolve all problems. Others felt the approach enabled more in-depth, less rushed consultations, suggesting that this time investment may ultimately support more efficient and sustainable care. At the same time, the HCPs faced challenges of implementation in routine practice, including the need for training to adopt the PC-IC approach.

### 4.2. Comparison with existing literature

Our results are in line with other studies that suggest that consultations with a broad, open dialogue not only focusing on medical issues, are perceived positively by both HCPs and patients [20]. One of the key elements is being seen as a person instead of a patient, which also relates closely to the concept of empathy, a well-known essential element of person-centred care [21]. However, Lundy et al. [22] showed that HCPs may experience greater challenges in expressing empathy towards patients whose life circumstances are more distant from their own, like the LESS patient group in our study. This requires attention and awareness within training or supervision for HCPs. Structural constraints that hamper person-centred care and empathic engagement, such as time pressure and protocol-driven care, are also described in other studies [6,13]. Shared goal setting and decision-making were not always perceived by the patients in our study, in contrast to their HCPs. HCPs may struggle to define goals when confronted with multiple complex issues, particularly when these are non-medical. Traditional lifestyle goals often do not match the priorities of patients with LSES, who often face challenging life circumstances. For these individuals, priorities may lie elsewhere, such as regaining stability, managing stress or feeling heard and supported [6,8]. This aligns with previous research showing that GPs often overestimate the extent of shared decision-making with patients with limited health literacy, while observational analyses reveal that exploration of patients’ needs and preferences, as well as active patient engagement, occurs only to a limited degree [23]. Existing healthcare communication frameworks, such as the Calgary–Cambridge Guide [24], as well as realist reviews of what works in person-centred primary care [13] emphasise the importance of person-centred communication and shared understanding. The visual tool in this study appeared to contribute to this person-centred, holistic communication.

The findings suggest that the visual tool indeed supported patients in their communication with their primary care health provider. According to the patients and HCPs involved, it helped patients with chronic conditions and HCPs structure conversations, clarify concerns and needs and in that way it supported mutual understanding. These findings underline the importance of tailoring communication and support to patients’ individual capacities and needs. This also highlights the relational and communicative dimensions of person-centred care, in which empathy, accessible communication and professional support remain essential. Another explanation may be insufficient assessment of patients’ comprehension. When explanations are complex or characterised by medical words, patients may not fully understand their situation, leading to difficulties in interpreting the relevance of specific choices, feasible treatment options or the goals discussed. As a result, HCPs may perceive the process as one of shared decision-making, while patients experience it as predominantly unidirectional, given their limited capacity to participate actively. In addition, research showed that people with limited health skills are less likely to engage actively in goal setting and shared decision-making. Such patients often express trust in professional authority (e.g. stating that ‘the doctor knows best’), which may be misconstrued as agreement, while in fact reflecting passivity and a perceived lack of agency. Like in the patient with claudication (Section 3.2.2 first quote) [25,26].

A discrepancy was observed between HCPs and patients regarding the use of the PC-approach with patients remembering little of a shift in the way they received chronic care. An explanation could be that when HCP implement small behavioural changes – such as asking more open-ended questions, summarising or listing options – it may feel like a different way of working to them, however patients see this as normal care [27].

In order to improve management of chronic diseases in patients with LSES, it is important to address social problems, which can exacerbate chronic diseases and decrease the ability for effective self-management and healthy lifestyle [28,29]. This study suggests that the visual tool to support communication encouraged the discussion of these social topics and that both HCP and patients appreciated this.

The positive experiences reported by both HCPs and patients with the PC-IC approach suggest that it may also offer a potential response to the so-called the ‘inverse care law’ [30]. This law, first described by Tudor Hart [31], posits that those populations with the greatest health needs often have the least access to appropriate and quality care. Patients with limited health literacy and lower education are overrepresented in chronic care [32,33]. By fostering stronger relationships, addressing patients’ social as well as medical contexts, and improving continuity of care, the PC-IC approach could help reducing inequity. Starting with a person-centred conversation may provide greater insight into the patient’s social context and treatment or advice will be better tailored to the patient’s capabilities and skills. As a result, patients with LSES may receive care that is better aligned with their situation, potentially contributing to more appropriate and effective support.

### 4.3. Strengths and limitations

A strength of this study is the dual perspective of both HCPs and patients about their experiences and insights of the PC-IC approach, especially about the conversation using the visual tool. In addition, the involvement of LSES patients who are often underrepresented in scientific research, constitutes a valuable contribution. The respondents (HCPs and patients) were from different regio’s in the Netherlands and also from urban and semi-rural areas which enhances the transferability of the findings to other general practices in the Netherlands.

Face-to-face interviews with patients from lower socioeconomic backgrounds enable the establishment of personal contact, the building of trust and a more nuanced understanding of their experiences. This is particularly important as written questionnaires or digital methods are often less effective among individuals with limited literacy skills. As a consequence, such interviews can provide richer and more reliable data from a group that would otherwise be underrepresented.

At the same time, this approach may be a limitation, as socially desirable responses could occur; patients might be inclined to give answers they believe the interviewer expects or wants to hear. We sought to minimise this by emphasising the importance of sharing genuine experiences and ensuring the confidentiality of participants’ responses. Additionally, only patients from the intervention group who were willing to participate were interviewed, which may have introduced self-selection bias. As this study focused on participants’ experiences and perceptions, the findings do not provide direct insight into communication dynamics during consultations.

This study included patients with LSES, but all researchers involved were highly educated. The interpretation of interviews of the patients may have been biased, given the researchers’ backgrounds and differences in living context. The researchers may also have been biased towards a more positive attitude regarding the newly implemented approach, as some members of the research team were involved in the development of this approach.

### 4.4. Implications for research and practice

Further research should focus on the larger-scale implementation of this person-centred approach in general practice. It is important to evaluate the effect over a longer period of time, both in terms of health outcomes and processes, such as shared goal setting and decision-making. In addition, it may be interesting to explore whether this approach is suitable in other domains, such as the social domain, for other HCPs in primary care as well as in specialist care. Furthermore, practical implications should be considered, including the need for organisational support, facilitation of interprofessional collaboration and sufficient time for patient encounters.

It may be useful to examine whether this PC-IC approach can be incorporated into healthcare education curricula. When future HCPs better understand the complexity of the accumulation of factors and what it means to live in less favourable (social) circumstances, they are better prepared to provide person-centred care. This enables them to explicitly consider health literacy, social determinants of health and the importance of shared decision-making in their practice.

## 5. Conclusion

This study explored the experiences of HCPs and patients with a PC-IC approach for LSES patients. Both groups considered the PC-IC approach valuable to broaden the scope of consultations, not only in terms of medical symptoms, but also in terms of more holistic and non-medical conversations. The HCPs experienced greater job satisfaction, and patients were more involved in the conversation. The approach resulted in care more tailored to the needs of the patient.

## Supplementary Material

Supplementary files.docx

## Appendix 2. Interview guide for healthcare providers (GP–PN)

### Introduction

Sign informed consent form

Start recording

Name:

Practice:

Role:

Date:

### General questions about the PC-IC programme

1. General understanding
  - What does the new way of working in chronic care (EMBOSS) entail for you (training, visual conversation tool, case-based meetings)?
  - What are you doing differently now compared before the project started?
  - Do you work more person-centred than before? Explanation.
  - Could you give a general description of how you applied EMBOSS (e.g. informing patients beforehand, sending/giving the visual conversation card, scheduling longer consultations)?
2. Use of the model and steps
  - Did you follow all seven steps of the model, or only some? Why? Explanation.
3. Applicability
  - Did you apply EMBOSS only with included patients, or also with others? With which ‘types’ of patients, and why?
4. Outcomes and perceived effects
  - What does this way of person-centred working bring?
    - For you as a healthcare professional?
      (Workload? Job satisfaction? Collaboration?)
    - For the patient?
      What did you notice, and which component contributed most?
      Does it benefit all patients equally? Why (not)?
  - Do you feel you get further with certain patients?
  - Does the organisation benefit?
5. Changes in consultations
  - Has the consultation changed compared to before? How?
  - Did you gain more insight into the patient’s context? Examples?
  - Did you refer more often? To whom?
  - Were these partners already known to you?

### Specific questions about the ‘EMBOSS programme’

1. Fit with needs of patients with low SES
  1. Which components did or did not match the needs of patients with low SES, and why?
  2. How does this compare with disease-specific care they previously received?
  3. Is this approach accessible and understandable for everyone?
2. Conducting a (broad) person-centred conversation
  - The EMBOSS model begins with exploring someone’s health and daily life through a broad conversation, which is often a different way of working. How did you experience this?
  - How did you conduct the conversation? Any example case?
  - Did you use the conversation card or any other tools? Why or why not?
3. Insights from the broad conversation
  - Did it provide insight into social context?
  - Did you learn new things about other life domains (e.g. financial issues, stress and debt)?
  - What did you learn about social determinants?
4. Handling multiple topics
  - Multiple domains may come up. What is your experience, and how do you deal with this?
5. Feeling equipped
  - Did you feel adequately equipped (training, conversation card, case discussions and network support)? Why or why not?
  - What else do you need?
  - Tips for colleagues?
6. Time
  - How much time did the first broad conversation usually take?
7. Use of the conversation tool
  - What is your experience using the conversation card?
  - How do you use it?
  - Did you send it beforehand? Why/why not?
  - With which patients do you use it?
  - Are any images/topics missing or unnecessary?
  - Tips for colleagues who find it difficult to use?
8. Goal setting
  - Does this approach help with setting goals?
  - Who determines goals or interventions?
  - Does it help patients think along and participate in decisions?
  - Did this lead to more referrals? To whom?
  - When do you revisit goals?
  - Do you feel prepared to set goals beyond health-related ones (e.g. stress, loneliness and debt)?
9. Care plan
  - Did you create a care plan?
    - How was it shared (digital, printed and record)?
  - If not, why not?
10. Evaluation/support
  - Was the final step – evaluating and supporting goals – reached with (some) patients? Tell us more.

### Shared decision-making/patient autonomy

1. Does the new programme enable better shared decision-making with low SES patients? How? Example?
2. Do you notice more confidence or autonomy among patients?
3. What changes do you observe in skills or behaviours
  (interaction, confidence, asking questions, preparation, involvement, etc.)?

### Collaboration and referrals

1. Has the new programme led to more referrals to other professionals or domains?
2. Were these different from usual?
3. Which professionals/domains (social domain, debt, lifestyle interventions, migrant organisations, community centre, etc.)?
4. Do you refer differently for low SES versus high SES patients?
5. Have new collaborations emerged? Regional or neighbourhood level?
6. How do you maintain collaborations?
7. How did referrals take place (warm transfer, in-house and neighbourhood-based)?
8. How well does neighbourhood collaboration align with the needs and possibilities of low SES patients?
9. Is there now a central care coordinator for patients with multiple chronic conditions?
10. What do you still need to build networks and collaboration?
11. What is the role of the regional care group?
12. Did the interprofessional collaboration training help you? How?

### Job satisfaction [database]

1. Has the programme contributed to job satisfaction? How?
2. What increases or decreases job satisfaction?
3. Which components contribute to this?

### Implementation [mismatch]

1. Now that the study has ended in your practice, how will you proceed? Continue or stop?
2. How was it to involve low SES/limited health literacy patients in the pilot (identifying, including, motivating and retaining)?
3. Did the programme provide adequate tools for recognising and communicating with low SES patients?
4. What conditions are needed for further implementation?
5. What skills/materials would you still like to learn or develop to better support low SES or complex patients (culturally sensitive care, collaboration, networking and intervision)?

### Closure

1. Are there any other issues related to the programme or low SES patients that we have not discussed but that you would like to mention?
2. Possible request for contribution to a Dutch professional journal if the interview is valuable.

End of interview. Thank you very much for your participation.

## Appendix 3. Interview guide for patients

### Introduction

Review, explain and sign the consent form.

Start recording.

### General

1. In the past year, have you visited your GP or the practice nurse [name] for your diabetes/COPD/CVRM and if so, how many times?

2. Do you feel that the care you received from your GP or practice nurse over the past year (for your diabetes/COPD/CVRM) was different from before?

- If yes: what was different (ask general here; we will go into more detail later in the interview)?
  - What did you find pleasant or positive about this?
  - And what did you find less pleasant or negative?
  - How did you feel about taking part in this?
- If no: ask whether there was, for example, a different type of conversation about daily life, concerns, goals, etc.

3. What information or explanation where you given beforehand about this new way of providing care? Do you remember?

4. Does this mean that you visited the practice nurse or GP more often in the past year for your chronic condition than before? What is the reason for that?

### Focus on having a person-centred conversation

5. Did you have a different type of conversation with the GP or practice nurse than before? Did the conversation cover more topics than just your illness (we also call this the broader person-centred conversation)?

6. How do you feel about talking about topics other than just your condition, such as diabetes or COPD?

- Did this lead to a different conversation than before? What was different? What did you think of that? And how long did the conversation last?
- Do you feel that this made you feel more seen or heard? What made you feel that way, or what did you notice? Can you give an example?
- What topics were discussed? And had you talked about this before? Can you give an example?
- Was it easier (or similar) for you to talk about certain topics compared to before? And why?
- Which topics does the practice nurse or GP now know more about than before? What do you think about that? Can you give an example?
- Do you feel that you can discuss more now than before? And what makes you feel that way?
- Do you feel that you can ask more questions than before? Could you give some examples?

### Materials

7. You just mentioned the broader conversation. Did the practice nurse or GP use any materials during that conversation, for example a conversation card with images? Or something else? And what exactly?

(First let them answer, then place the conversation card (or spiderweb tool) on the table.)

8. Do you recognise this conversation card?

- Did the practice nurse use this during the consultation?
  - If not: was something else used? What exactly?

9. How did you experienced the conversation with your practice nurse or GP when using a conversation card (or another tool, if that comes up) with images? And why?

- Where you given the conversation card beforehand or sent it home?
- By having the conversation using this card with pictures, did it lead to a different conversation than before? And why was that? Did you find it pleasant, easier, or less pleasant/negative?
- What do you think about using images or pictures in conversations with the practice nurse?
- Are there any topics missing from the card?
- Are there any images that are unclear or that we should change?

### Discussing different topics/understanding the social context

10. The practice nurse asks about more than just your illness or symptoms. She also asks questions about, for example, your home situation, your work, or simply how you are doing. What do you think about that?

11. During such a broader conversation (using the conversation card), problems, worries, or complaints may have come up – [refer to topics the participant already mentioned] – that you had not talked about in this way before. What effect does that have on your health? Does it influence your health in any way?

For example: that the conversation now includes stress or other types of social problems, and what effect that has on their health, or whether they themselves start to see the link between physical complaints and social issues.

- Do you think it is important that the practice nurse [name] also knows more about your life? Why or why not?
- Is this different from the care you received before this study?

12. Were any (treatment) goals set? What did you think of that?

- Was the process of setting goals or making a treatment plan different from before this study?
- Do these goals or recommendations suit you well, and why?
- Are the goals or recommendations about health (your illness), or were other types of goals also agreed on (for example: goals related to social issues, social work, loneliness, debt, well-being, etc.)?
- Could you give some examples?
- Are you able to conduct or follow these goals or tips? Why or why not (think also of skills and practical possibilities: costs, availability nearby, outside working hours, language, cultural relevance, etc.)?
- Who created these goals? What was your own role in setting them?
- About the goals/treatment/recommendations:
  - Where were these goals written down? Or is there somewhere you can view them?
  - Did you receive any information on paper or online to help you achieve your goals? What kind of information? And what did you think of it?
    (If not: What kind of information would you have liked?)
- Have you been able to take these goals home? Would you like to?
- Did setting these goals give you confidence that you would be able to achieve them? Why or why not?
- What would help increase your confidence?

13. Do you now know more, or better, what you can do yourself? If yes, what exactly?

(For example: following advice, recognising complications, taking medication, understanding your condition.)

14. Did you receive other advice, or were you referred to another care or support professional?

If yes, to whom? And is this different from before?

- How did the referral go? Was it a warm handover?
- Are you currently seeing several care or support providers? Which ones
   (also check for social domain support)?

15. In what other ways did the healthcare professional help you to achieve your goals (support) ?

16. You set several goals together with your GP or practice nurse, which also resulted in recommendations. Was it possible for you to achieve these goals?

- If yes: was this discussed or evaluated with the practice nurse or GP?
  (Think of step 7: Evaluation in the model.)
- If no: why not? Was it maybe not possible for you, maybe too difficult, not a good fit, etc?
- Would you have preferred another goal, or to do something differently? And what would that be? Have you discussed that with your HCP?

### Healthcare provider

17. How is your contact with your HCP?

18. Do you feel that, because of the new care programme (the broader conversation), you now have better or different contact with your practice nurse or GP? Could you explain that or give an example?

19. Do you feel that the practice nurse or GP understands you well? Why or why not? Has this changed because of the new care programme? In what way?

20. Does the practice nurse or GP explain things clearly to you? Why or why not? Are many difficult words used? Has this changed because of the new care programme? If so, how?

21. Do you understand the GP or practice nurse? For example, when you get home, do you still know what you need to do, or what the GP or practice nurse has told you?

22. Do you feel that you can ask or tell the practice nurse everything? Why? Has this changed because of the new care programme? In what way?

23. [Only if referred after the broader conversation] You mentioned earlier that you were referred to another HCP. Do you feel that they work well together? What makes you think that?

24. Is there anything else you would like to share about this new way of providing care that I have not asked about?

25. And what questions do you have for me? I am sure there is something I forgot to ask.

Finally, we have a few short general questions:

(Probably not needed, as you already have this information)

- Occupation:
- Completed education:
- Age:
- Number of years receiving care from the HCP:
- Place of birth:
