# Supplementary material for: Person-centred integrated primary care targeting patients with chronic conditions and low socioeconomic status: an interview study exploring experiences from healthcare providers and patients
Source: Scand J Prim Health Care. 2026 Oct 29;44(1):2734060. doi: 10.1080/02813432.2026.2734060 (PMC13721832; doi:10.1080/02813432.2026.2734060)
Supplement: Supplementary file 1 [file IPRI_A_2734060_SM0094.docx]

**Supplementary files**

Appendix 1. Schematic representation of person-centred and integrated care (PC-IC) in seven steps. Also with tips for healthcare providers and their organisations.

Appendix 2. Interview Guide for Healthcare Providers (GP -PN)

**Introduction**

Sign informed consent form

Start recording

Name:

Practice:

Role:

Date:

**General questions about the PC-IC programme**

1. General understanding

- What does the new way of working in chronic care (EMBOSS) entail for you (training, visual conversation tool, case-based meetings)?
- What are you doing differently now compared before the project started?
- Do you work more person-centred than before? Explanation.
- Could you give a general description of how you applied EMBOSS?
  *(e.g., informing patients beforehand, sending/giving the visual conversation card, scheduling longer consultations)*

2. Use of the model and steps

- Did you follow all seven steps of the model, or only some? Why? Explanation.

3. Applicability

- Did you apply EMBOSS only with included patients, or also with others? With which ‘types’ of patients, and why?

4. Outcomes and perceived effects

- What does this way of person-centred working bring?
  - For you as a healthcare professional?
    (Workload? Job satisfaction? Collaboration?)
  - For the patient?
    What did you notice, and which component contributed most?
    Does it benefit all patients equally? Why (not)?
- Do you feel you get further with certain patients?
- Does the organisation benefit?

5. Changes in consultations

a. Has the consultation changed compared to before? How?
b. Did you gain more insight into the patient’s context? Examples?
c. Did you refer more often? To whom?
d. Were these partners already known to you?

**Specific questions about the ‘EMBOSS programme’**

1. Fit with needs of patients with low SES
1. Which components did or did not match the needs of patients with low SES, and why?
2. How does this compare with disease-specific care they previously received?
3. Is this approach accessible and understandable for everyone?
2. Conducting a (broad) person centred conversation
a. The EMBOSS model begins with exploring someone’s health and daily life through a broad conversation, which is often a different way of working. How did you experience this?
b. How did you conduct the conversation? Any example case?
c. Did you use the conversation card or any other tools? Why or why not?
3. Insights from the broad conversation
a. Did it provide insight into social context?
b. Did you learn new things about other life domains (e.g., financial issues, stress, debt)?
c. What did you learn about social determinants?
4. Handling multiple topics
a. Multiple domains may come up. What is your experience, and how do you deal with this?
5. Feeling equipped
a. Did you feel adequately equipped (training, conversation card, case discussions, network support)? Why or why not?
b. What else do you need?
c. Tips for colleagues?
6. Time
a. How much time did the first broad conversation usually take?
7. Use of the conversation tool
a. What is your experience using the conversation card?
b. How do you use it?
c. Did you send it beforehand? Why/why not?
d. With which patients do you use it?
e. Are any images/topics missing or unnecessary?
f. Tips for colleagues who find it difficult to use?
8. Goal Setting
a. Does this approach help with setting goals?
b. Who determines goals or interventions?
c. Does it help patients think along and participate in decisions?
d. Did this lead to more referrals? To whom?
e. When do you revisit goals?
f. Do you feel prepared to set goals beyond health-related ones (e.g., stress, loneliness, debt)?
9. Care Plan
a. Did you create a care plan?
   - How was it shared? (digital, printed, record)
b. If not, why not?
10. Evaluation/Support
    a. Was the final step—evaluating and supporting goals—reached with (some) patients? Tell us more.

**Shared decision-making / patient autonomy**

1. Does the new programme enable better shared decision-making with low SES patients? How? Example?
2. Do you notice more confidence or autonomy among patients?
3. What changes do you observe in skills or behaviours?
   *(interaction, confidence, asking questions, preparation, involvement, etc.)*

**Collaboration and referrals**

1. Has the new programme led to more referrals to other professionals or domains?
2. Were these different from usual?
3. Which professionals/domains (social domain, debt, lifestyle interventions, migrant organisations, community centre, etc.)?
4. Do you refer differently for low SES versus high SES patients?
5. Have new collaborations emerged? Regional or neighbourhood level?
6. How do you maintain collaborations?
7. How did referrals take place (warm transfer, in-house, neighbourhood-based)?
8. How well does neighbourhood collaboration align with the needs and possibilities of low SES patients?
9. Is there now a central care coordinator for patients with multiple chronic conditions?
10. What do you still need to build networks and collaboration?
11. What is the role of the regional care group?
12. Did the interprofessional collaboration training help you? How?

**Job satisfaction**

1. Has the programme contributed to job satisfaction? How?
2. What increases or decreases job satisfaction?
3. Which components contribute to this?

**Implementation**

1. Now that the study has ended in your practice, how will you proceed? Continue or stop?
2. How was it to involve low SES/limited health literacy patients in the pilot?
   *(identifying, including, motivating, retaining)*
3. Did the programme provide adequate tools for recognising and communicating with low SES patients?
4. What conditions are needed for further implementation?
5. What skills/materials would you still like to learn or develop to better support low SES or complex patients?
   *(culturally sensitive care, collaboration, networking, intervision)*

**Closure**

1. Are there any other issues related to the programme or low SES patients that we have not discussed but that you would like to mention?
2. Possible request for contribution to a Dutch professional journal if the interview is valuable.

End of interview. Thank you very much for your participation.

Appendix 3. Interview Guide for Patients

Introduction

Review, explain, and sign the consent form.

Start recording.

**General**

1. In the past year, have you visited your GP or the practice nurse [name] for your diabetes/COPD/CVRM, and if so, how many times?
2. Do you feel that the care you received from your GP or practice nurse over the past year (for your diabetes/COPD/CVRM) was different from before?
   a. *If yes:* What was different? (Ask general here; we will go into more detail later in the interview).
   i. What did you find pleasant or positive about this?
   ii. And what did you find less pleasant or negative?
   iii. How did you feel about taking part in this?
   b. *If no:* Ask whether there was, for example, a different type of conversation about daily life, concerns, goals, etc.
3. What information or explanation where you given beforehand about this new way of providing care? Do you remember?
4. Does this mean that you visited the practice nurse or GP more often in the past year for your chronic condition than before? What is the reason for that?

**Focus on having a person-centred conversation**

5. Did you have a different type of conversation with the GP or practice nurse than before? Did the conversation cover more topics than just your illness?
   (We also call this the *broader person-centred conversation*)
6. How do you feel about talking about topics other than just your condition, such as diabetes or COPD?
   a. Did this lead to a different conversation than before? What was different? What did you think of that? And how long did the conversation last?
   b. Do you feel that this made you feel more seen or heard? What made you feel that way, or what did you notice? Can you give an example?
   c. What topics were discussed? And had you talked about this before? Can you give an example?
   d. Was it easier (or similar) for you to talk about certain topics compared to before? And why?
   e. Which topics does the practice nurse or GP now know more about than before? What do you think about that? Can you give an example?
   f. Do you feel that you can discuss more now than before? And what makes you feel that way?
   g. Do you feel that you can ask more questions than before? Could you give some examples?

**Materials**

7. You just mentioned the broader conversation. Did the practice nurse or GP use any materials during that conversation, for example a conversation card with images? Or something else? And what exactly?
   *(First let them answer, then place the conversation card (or spiderweb tool) on the table.)*
8. Do you recognise this conversation card?
   a. Did the practice nurse use this during the consultation?
   i. If not: Was something else used? What exactly?
9. How did you experienced the conversation with your practice nurse or GP when using a conversation card (or another tool, if that comes up) with images? And why?
   a. Where you given the conversation card beforehand or sent it home?
   b. By having the conversation using this card with pictures, did it lead to a different conversation than before? And why was that? Did you find it pleasant, easier, or less pleasant/negative?
   c. What do you think about using images or pictures in conversations with the practice nurse?
   d. Are there any topics missing from the card?
   e. Are there any images that are unclear or that we should change?

**Discussing different topics / understanding the social context**

10. The practice nurse asks about more than just your illness or symptoms. She also asks questions about, for example, your home situation, your work, or simply how you are doing. What do you think about that?
11. During such a broader conversation (using the conversation card), problems, worries, or complaints may have come up — [refer to topics the participant already mentioned] — that you had not talked about in this way before. What effect does that have on your health? Does it influence your health in any way?

*For example: that the conversation now includes stress or other types of social problems, and what effect that has on their health, or whether they themselves start to see the link between physical complaints and social issues.*

a. Do you think it is important that the practice nurse [name] also knows more about your life? Why or why not?
b. Is this different from the care you received before this study?

12. Were any (treatment) goals set? What did you think of that?
    a. Was the process of setting goals or making a treatment plan different from before this study?
    b. Do these goals or recommendations suit you well, and why?
    c. Are the goals or recommendations about health (your illness), or were other types of goals also agreed on?
    *(For example: goals related to social issues, social work, loneliness, debt, wellbeing, etc.)* d. Could you give some examples?
    e. Are you able to conduct or follow these goals or tips? Why or why not?
    *(Think also of skills and practical possibilities: costs, availability nearby, outside working hours, language, cultural relevance, etc.)*
    f. Who created these goals? What was your own role in setting them?
    g. About the goals / treatment / recommendations:
    - Where were these goals written down? Or is there somewhere you can view them?
    - Did you receive any information on paper or online to help you achieve your goals? What kind of information? And what did you think of it?
    *(If not: What kind of information would you have liked?)*
    h. Have you been able to take these goals home? Would you like to?
    i. Did setting these goals give you confidence that you would be able to achieve them? Why or why not?
    j. What would help increase your confidence?
13. Do you now know more, or better, what you can do yourself? If yes, what exactly?
    *(For example: following advice, recognising complications, taking medication, understanding your condition.)*
14. Did you receive other advice, or were you referred to another care or support professional?
    If yes, to whom? And is this different from before?
    a. How did the referral go? Was it a *warm handover*?
    b. Are you currently seeing several care or support providers? Which ones?
    *(Also check for social domain support)*
15. In what other ways did the healthcare professional help you to achieve your goals?
    *(Support)*
16. You set several goals together with your GP or practice nurse, which also resulted in recommendations. Was it possible for you to achieve these goals?
    a. If yes: Was this discussed or evaluated with the practice nurse or GP?
    *(Think of step 7: Evaluation in the model.)*
    b. If no: Why not? Was it maybe not possible for you, maybe too difficult, not a good fit, etc?
    c. Would you have preferred another goal, or to do something differently? And what would that be? Have you discussed that with your HCP?

**Healthcare provider**

17. How is your contact with your HCP?
18. Do you feel that, because of the new care programme (the broader conversation), you now have better or different contact with your practice nurse or GP? Could you explain that or give an example?
19. Do you feel that the practice nurse or GP understands you well? Why or why not? Has this changed because of the new care programme? In what way?
20. Does the practice nurse or GP explain things clearly to you? Why or why not? Are many difficult words used? Has this changed because of the new care programme? If so, how?
21. Do you understand the GP or practice nurse? For example, when you get home, do you still know what you need to do, or what the GP or practice nurse has told you?
22. Do you feel that you can ask or tell the practice nurse everything? Why? Has this changed because of the new care programme? In what way?
23. [Only if referred after the broader conversation] You mentioned earlier that you were referred to another HCP. Do you feel that they work well together? What makes you think that?
24. Is there anything else you would like to share about this new way of providing care that I have not asked about?
25. And what questions do you have for me? I am sure there’s something I forgot to ask.

Finally, we have a few short general questions:

(Probably not needed, as you already have this information)

- Occupation:
- Completed education:
- Age:
- Number of years receiving care from the HCP:
- Place of birth:
